# Age- and Sex-Associated Wnt Signaling Dysregulation is Exacerbated from the Early Stages of Neuropathology in an Alzheimer’s Disease Model

**DOI:** 10.1007/s11064-024-04224-7

**Published:** 2024-08-21

**Authors:** Elizabeth Colín-Martínez, César Espino-de-la-Fuente, Clorinda Arias

**Affiliations:** Departamento de Medicina Genómica y Toxicología Ambiental, Instituto de Investigaciones Biomédicas, Universidad Nacional Autónoma de México, Ciudad de México, 04510 México

**Keywords:** Wnt signaling, Aging brain, Alzheimer’s disease, Dkk-1, Phospho-tau, Sex differences

## Abstract

Emerging studies suggest that Wnt signaling is dysregulated in the brains of AD patients, suggesting that this pathway may also contribute to disease progression. However, it remains to be determined whether alterations in the Wnt pathway are the cause or consequence of this disease and which elements of Wnt signaling mainly contribute to the appearance of AD histopathological markers early in disease compared to what occurs during normal aging. The present study aimed to describe the status of several canonical Wnt pathway components and the expression of the AD marker p-tau in the hippocampi of female and male 3xTg-AD mice during disease progression compared to those during normal aging. We analyzed the levels of the canonical Wnt components Wnt7a, Dkk-1, LRP6 and GSK3β as well as the levels of p-tau and BDNF at 3, 6, 9–12 and 18 months of age. We found a gradual increase in Dkk-1 levels during aging prior to Wnt7a and LRP5/6 depletion, which was strongly exacerbated in 3xTg-AD mice even at young ages and correlated with GSK3β activation and p-tau-S202/Thr205 expression. Dkk-1 upregulation, as well as the level of p-tau, was significantly greater in females than in males. Our results suggest that Dkk-1 upregulation is involved in the expression of several features of AD at early stages, which supports the possibility of positively modulating the canonical Wnt pathway as a therapeutic tool to delay this disease at early stages.

## Introduction

Alzheimer’s disease (AD) is the most common age-related dementia [1]. Pathologically, it is characterized by the accumulation of extracellular amyloid plaques (formed of Aβ peptides) and intracellular neurofibrillary tangles (made of p-tau) [2, 3] as well as the progressive loss of synapses and neurons [4]. The etiology of this disease has not been fully determined; however, emerging studies have reported a role for suppressed Wnt/β-catenin signaling in both experimental models and AD patients [5–8]. In the adult brain, the Wnt pathway is crucial for the maintenance of neuronal morphology and synaptic connectivity, and its dysregulation is also involved in Aβ production and tau phosphorylation [9–12]. Wnt agonists consist of 19 secreted glycoproteins that activate different receptors and coreceptors of the frizzled (FZD) family and the low-density lipoprotein receptor-related proteins 5 and 6 (LRP5/6) in a context- and age-dependent manner. Interestingly, genetic variants of LRP6 have been linked to late-onset AD [13, 14] as well as reduced FZD1 and FZD7 in the brain in the early stages of AD [15]. The canonical Wnt pathway is inhibited by endogenous antagonists, such as Dickkopf proteins (Dkk-1-3), which antagonize the receptors LRP5/6; Wnt inhibitory protein (WIF-1), which binds to Wnt agonists; and related soluble proteins, such as Frizzled (sFRP1-5), which acts on both Wnt agonists and FZD receptors [16, 17]. Activation of the canonical Wnt signaling cascade prevents the degradation of β-catenin, which translocate into the nucleus to activate the TCF/LEF (T-cell factor/lymphoid enhancing factor family) transcription factor family, promoting the transcription of genes involved in neuronal plasticity, memory, and learning [18–22]. The canonical agonist Wnt7a and the canonical antagonist Dkk-1 have attracted special interest because of their presence throughout life in the mature brain and their positive or negative role in regulating brain plasticity (reviewed by [23–25]. During aging, significant decreases in the expression of the Wnt7a/b, Wnt2b, Wnt6, FZD2, and FZD3 receptors and increases in the levels of the negative regulators sFRP1 and GSK3β were found in the prefrontal cortex of aged humans [7]. Similarly, different changes in Wnt agonist and antagonist have been described in the hippocampus, cerebellum and cerebral cortex of aged rats [26] and in the long-lived rodent Octodon degus [27]. Alterations in several components of the Wnt pathway have also been described in the brains of AD patients. A significantly high content of Dkk-1 was reported in neurons that also carried p-tau in the temporal cortex [28], as was a decreased transcription of the antagonist *WIF-1* [29]. Moreover, proteomic analysis of the brains of AD patients revealed the participation of Wnt signaling as one of the molecular networks particularly affected in this disease [30]. Currently, the progression of changes in several components of the Wnt pathway during the course of this disease in the 3xTg-AD mouse model and how these changes occur during the natural aging process in females and males are unclear. Thus, the aim of this study was to analyze the activation status of the canonical Wnt pathway throughout the progression of AD-like pathology and nonpathological aging in the hippocampi of 3xTg-AD and WT female and male mice through age. Our results showed early upregulation of the canonical Wnt antagonist Dkk-1, accompanied by overactivation of GSK3β correlates with to the occurrence of tau hyperphosphorylation. Dkk-1 upregulation was more prominent in females than in males in both WT and transgenic mice. Although these changes were partially observed as part of the aging process in the WT mice, the 3xTg-AD mice exhibited exacerbated dysregulation of the analyzed Wnt components, providing additional information on the molecular alterations that occur during aging and AD.

## Materials and Methods

### Animals

WT C57BL/6 (male *n* = 12, female *n* = 16) and 3xTg-AD (male *n* = 14, female *n* = 29) at different ages: young (3 months old), mature (6 months old, expressing AD-related hallmarks), middle aged (9–12 months old) and old (18 months old) were used. Animals were handled with all precautions necessary to avoid suffering in accordance with the ARRIVE guidelines and the Rules for Research in Health Matters (Mexico) with the approval of the local Animal Care Committee. During the whole experimental procedure, 4–5 mice per cage were housed in a laboratory environment with a 12 h artificial light/dark cycle and free access to water and food. To verify the homozygosity of the female and male transgenic mice, genotyping was carried out using the following primers: APP-tau, 5tauRev (5’-TCCAAAGTTCACCTGATAGT-3’); APP internal (5’-GCTTGCACCAGTTCTGGATGG-3’); and Thy12.4 (5’-GAGGTATTCAGTCATGTGCT-3’). To amplify PS1, PS1-K13 (5’-CACACGCACACTCTGACATGCACAGGC-3’) and PS1-K15 (5’-AGGCAGGAAGATCACGTGTTCCAAGTAC-3’) were used.

### Western Blotting

The brains of 4–10 animals per group of each age and genotype were obtained after decapitation. The hippocampus was carefully dissected and homogenized in 200 µl of RIPA lysis buffer (50 mmol/L Tris-HCl, pH 7.5; 150 mmol/L NaCl; 1% NP-40; 0.5% DOC plus complete inhibitor cocktail from Roche Diagnostics, Indianapolis, IN, USA) and sonicated in 20-second pulses 3 times at 4 °C. The samples were centrifuged at 14,000 rpm for 15 min at 4 °C, after which the supernatants were collected. The protein concentration was determined using a DC Protein Assay (Bio-Rad, USA). The lysates were boiled in Laemmli buffer and separated via 12% SDS‒PAGE. The proteins were then transferred to PVDF membranes (Millipore, Ireland). The membranes were blocked with 5% nonfat milk/PBS and incubated with one of the following primary antibodies at 4 °C overnight in 5% nonfat milk/PBS: Dkk-1 (1:500; Abcam Cat #ab61275), Wnt7a/b (1:250; Abcam Cat #ab100792), tau p-S202/Thr205 (1:1000; Thermo Scientific Cat # MN1020), BDNF (1:250; Abcam Cat #ab203573), LRP6 (1:1000; Santa Cruz Biotechnology Cat #sc-25317), p-S9 GSK3β (1:1000 Cell Signaling Technology Cat #9336), p-Tyr216 GSK3β (1:1000; Bio-Rad Cat #AHP2627), GSK3β total (1:1000 Cell Signaling Technology Cat #9315). Next, the membranes were washed and incubated with horseradish peroxidase-conjugated secondary antibodies (1:10,000; anti-mouse IgG or anti-rabbit IgG conjugated with horseradish peroxidase; Santa Cruz Biotechnology) in 5% nonfat milk/PBS for 2 h at room temperature (RT). At the end of this period, the membranes were visualized using Immobilon Western Chemiluminescent HRP Substrate (Millipore, Billerica, MA, USA) on Kodak X-OMAT film. Densitometric analysis of the bands was performed with ImageJ software (NIH). The values ​​obtained for each band were normalized to the values obtained for the respective Ponceau-stained bands, which was used as a loading control. The results are expressed as arbitrary optical density units (ODUs).

### Immunofluorescence

Coronal Sect. (30 μm) of the hippocampus from 18-month-old WT and 3xTg-AD female mice (3 animals per condition) were obtained, washed 3 times with 0.1 M PB, permeabilized with 0.3% Triton X-100/0.1 M PB for 30 min, and incubated with blocking solution (1% horse serum/0.3% Triton X-100 in 0.1 M PB) for two hours at RT. The antibody for Dkk-1 was used at a concentration of 1:200, and the antibody for the MAP-2 (Abcam Cat# ab183830) protein was used at a dilution of 1:500 and incubated overnight at 4 °C for 24 h. At the end of this incubation, brain samples were washed 3 times for 10 min each with washing solution (0.3% Triton X-100/0.1 M PB) and then incubated with secondary antibodies conjugated to Alexa Fluor (Invitrogen, Carlsbad, CA, USA); for Dkk-1, 488 donkey anti-mouse (1:300) was used, and for MAP-2, 546 goat anti-rabbit (1:300) was used for MAP-2, 2 h at RT. At the end of this incubation, the hippocampal sections were washed and covered with mounting medium (DAKO, Carpinteria, S3023) and visualized via confocal microscopy (Nikon A1R + STORM).

### Statistical Analysis

GraphPad Prism 8.0 (GraphPad Software, Inc) and the results of the statistical analyses are presented as dot plots. All the data are expressed as the mean ± standard error (SEM). Assessment of data normality was evaluated using the Shapiro-Wilk test. Multiple comparisons among groups were performed by two- or three-way ANOVA followed by Tukey’s post hoc test. Pearson’s correlation, R values and P values, was measured to determine the relationship between Dkk-1 levels and p-tau. Differences were considered to be statistically significant when the p value < 0.05. The results are expressed as optical density units (O.D.U.).

## Results

### Changes in Canonical Wnt Components during Aging and in AD-Like Pathology

**Fig. 1:**
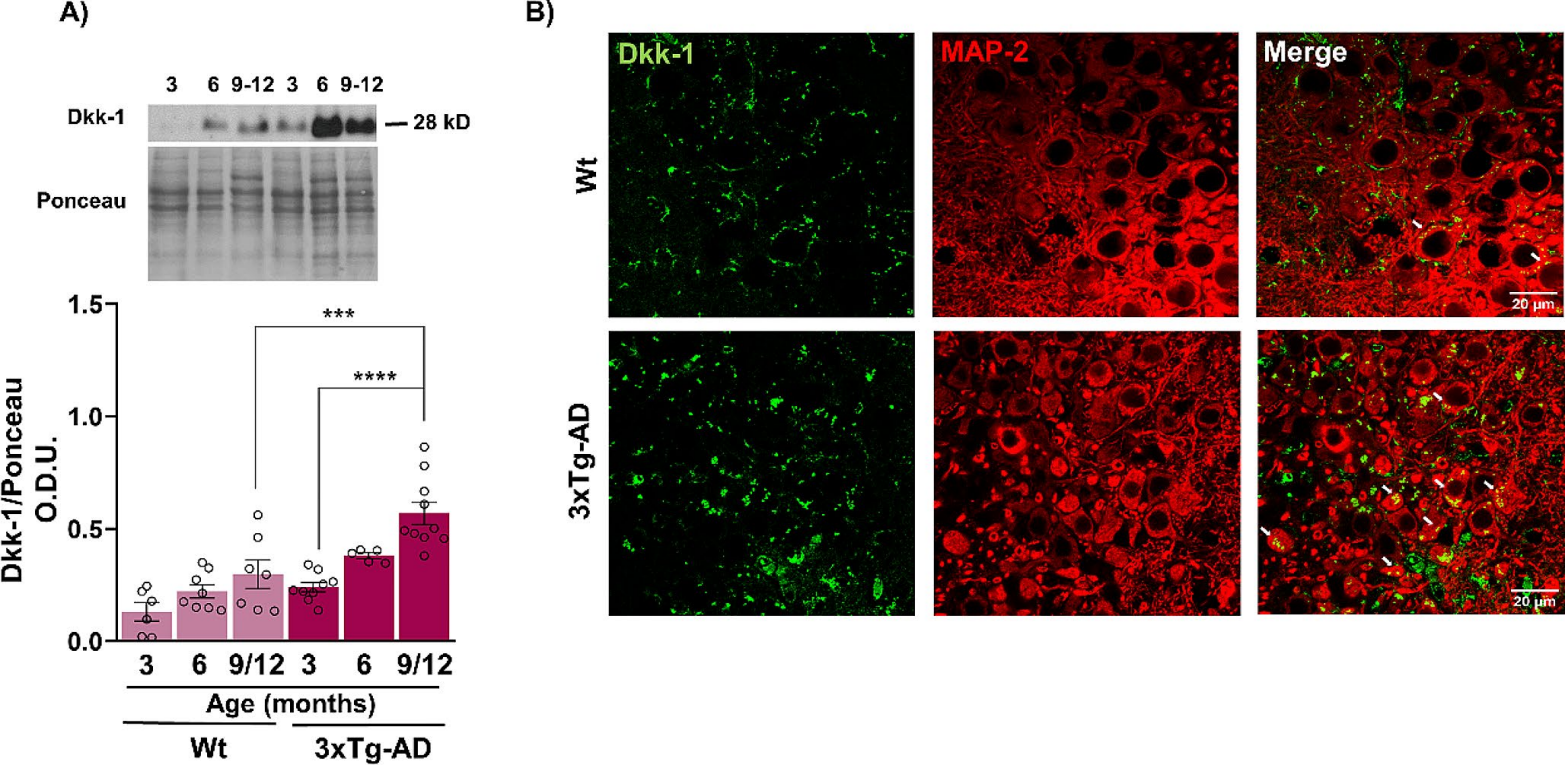
Changes in Dkk-1 protein levels in the hippocampus from female mice during aging and in the AD model. Representative Western blot and densitometric analysis of Dkk-1 from WT and 3xTg-AD female mice of different ages (**A**). Representative confocal images of the hippocampal CA3 region from 18-month-old WT and 3xTg-AD mice (**B**). In transgenic mice, more intense staining was observed, and clusters of Dkk-1 (stained in green) formed inside the neuronal somata (arrows) (stained in red with the MAP-2 antibody) (**B**). The bar graphs show the mean ± SEM from 5–10 animals per age group. Statistical significance was determined by two-way ANOVA (***p < 0.001, ****p < 0.0001). Scale bar = 20 μm

To determine the activation status of canonical Wnt signaling in the hippocampus during aging and throughout the progression of AD-type pathology, we analyzed the levels of the canonical antagonist and agonist Dkk-1 and Wnt7a in female WT (C57BL/6) and 3xTg-AD mice at 4 different ages: young (3 months old), mature (6 months old), middle aged (9–12 months old) and old (18 months old). As shown in Fig. 1, in female WT mice, Dkk-1 levels tended to increase with age, although they did not reach statistical significance. In contrast, in the hippocampus of 3xTg-AD female mice, Dkk-1 levels were significantly greater in middle-aged compared with young animals (F_1, 39_ = 25.80, *p* < 0.0001) and with transgenic versus WT animals (F_2, 39_ = 17.81 *p* < 0.001) (Fig. 1A). Interestingly, the significant increase in Dkk-1 at early ages in 3xTg-AD mice occurred in time with the appearance of the histopathological hallmarks of AD (increased Aβ and p-tau accumulation, cognitive deficit, and gliosis), as previously reported in this transgenic model [31]. Dkk-1 was found localized mainly in the hippocampal neuronal somata and particularly in the CA3 region in 18-month-old 3xTg-AD female mice compared to WT mice of the same age (Fig. 1B). In transgenic mice, more intense staining was observed, and clusters were formed inside the neuronal somata (Fig. 1B, arrows).

**Fig. 2:**
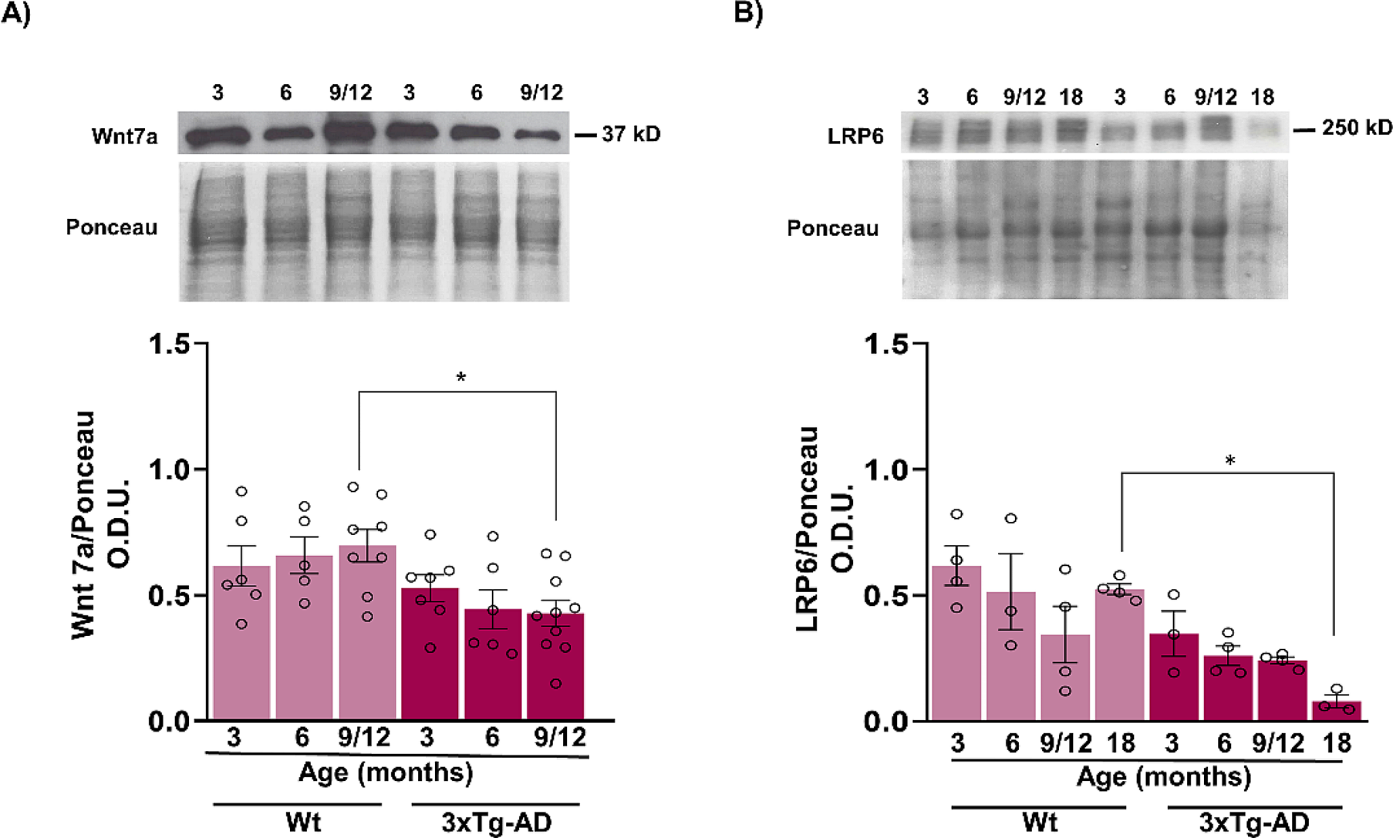
The protein levels of Wnt7a and LRP6 are decreased in 3xTg-AD female mice. The total Wnt7a concentration was significantly lower in middle-aged 3xTg-AD mice than in young WT mice (**A**). The LRP6 coreceptor was significantly reduced until 18 months of age in both WT and 3xTg-AD mice (**B**). The bar graphs show the mean ± SEM from 4–10 animals per age group. Statistical significance was determined by two-way ANOVA (*p < 0.05)

To analyze the balance between the effects of the Wnt antagonist and agonist, we measured the concentration of the agonist Wnt7a. In the case of female 3xTg-AD mice, low levels of Wnt7a were present during the ages tested, but only at middle-aged were significantly lower than those in the WT mice (F_1,36_ = 12.2, *p* < 0.05) (Fig. 2A). Given that a reduction in the Wnt canonical coreceptor, LRP6 has been associated with aging and the late onset of AD 13, the LRP6 protein level was quantified at different ages and during Alzheimer disease-like progression. A significant reduction of nearly 60% in the 3xTg-AD mice at 18 months compared with that in the WT mice at 18 months (F_1,21_ = 24.52, *p* < 0.05) (Fig. 2B). These results suggest that canonical Wnt signaling activation is compromised beginning in the early stage of AD-like pathology in the 3xTg-AD model, starting with the upregulation of Dkk-1 and, later, with a decrease in the expression of the Wnt7a agonist and the LRP6 coreceptor.

### GSK3β Activation and Tau Phosphorylation in 3xTg-AD Mice

**Fig. 3:**
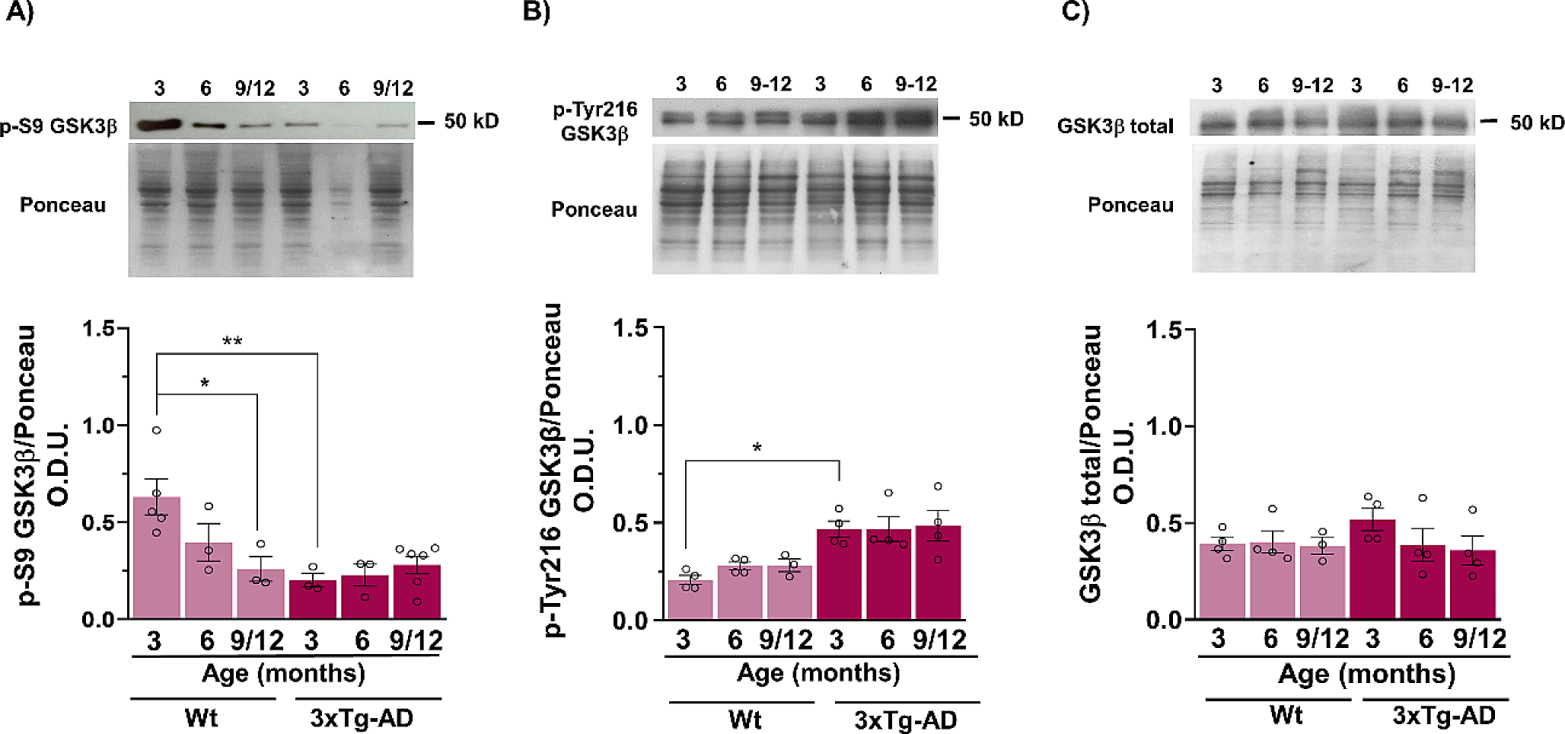
GSK3β is activated during aging and in the AD model. Representative Western blots and densitometry analysis results showing the protein levels of p-S9GSK3β (inhibitory residue) (**A**), p-Tyr216 (activation residue) (**B**) and total GSK3β (**C**). An age-dependentdownregulation of p-S9GSK3β was observed along with an upregulation of p-Tyr216 in WT animals. These changes occurred earlier in the 3xTg-AD female mice than in the control mice, but there were no significant changes in total GSK3β. The bar graphs show the mean ± SEM from at least 3–6 independent experiments. (*p < 0.05, **p < 0.01, two-way ANOVA)

To determine the involvement of the downstream regulator of Wnt signaling, the kinase GSK3β, we analyzed the active form of the enzyme through the measurement of the activation residue p-Tyr216-GSK3β and the inhibitory site p-S9-GSK3β. The aging of mice presented an activation profile of GSK3β, as revealed by a reduction in the p-S9 concentration and an increase in the p-Tyr216. A significant decrease in p-S9 was observed in WT animals compared young and middle-aged mice (F_2,17_ = 2.129, *p* < 0.05). This activation was exacerbated in the 3xTg-AD mice, which exhibited significant activation (F_1,17_ = 9.785, *p* < 0.01) beginning at 3 months before the appearance of AD markers and remained activated at all analyzed ages (Fig. 3A). A similar pattern of activation of GSK3β was observed through quantification of the p-Tyr216 site (F_1,17_ = 28.52, *p* < 0.05) (Fig. 3B). At all ages, the total kinase levels did not significantly change (Fig. 3C).

**Fig. 4:**
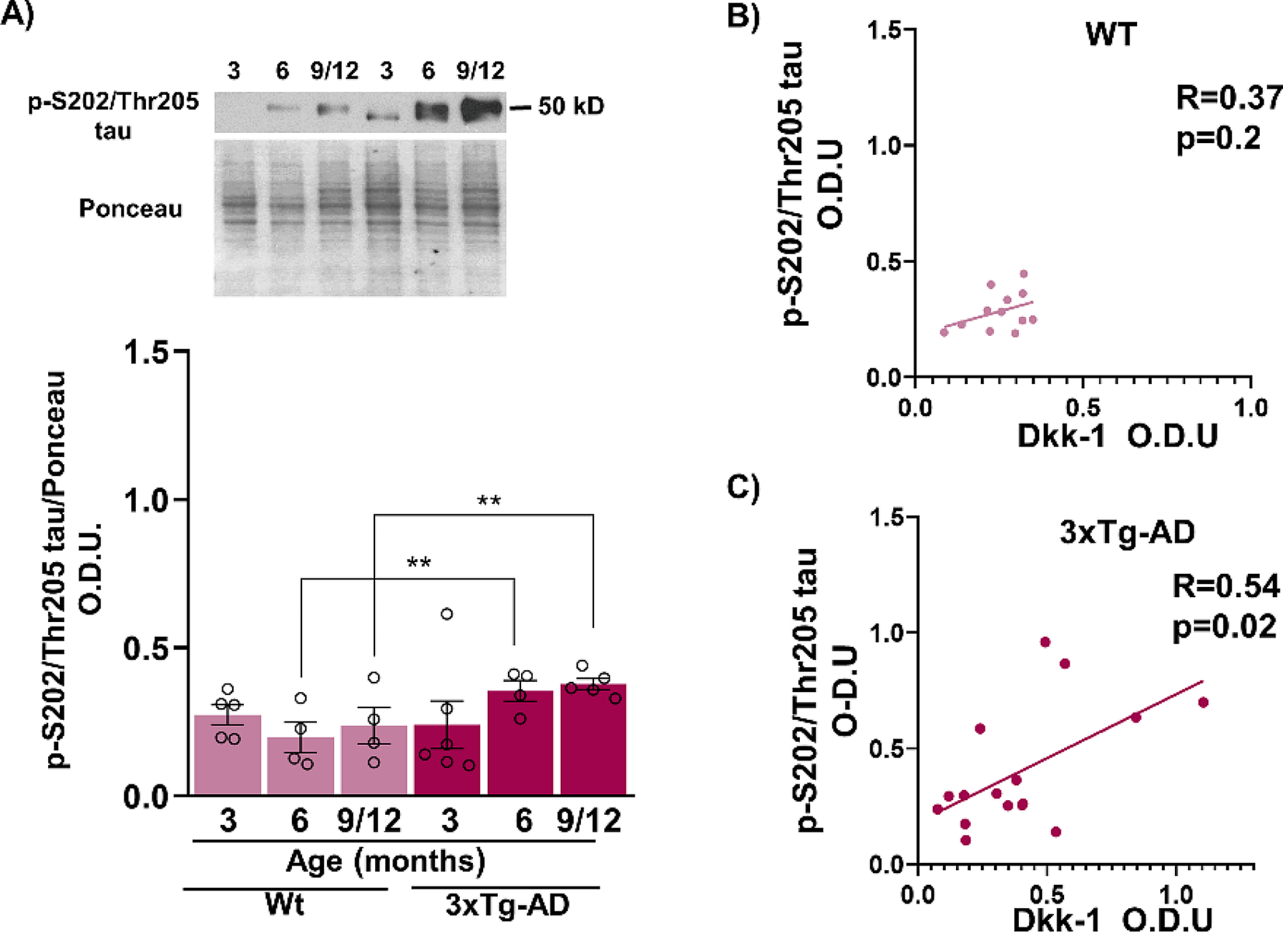
Age-dependent increase of tau phosphorylation in the 3xTg-AD female mice. A increase in p-S202/Thr205 tau was observed in 3xTg-AD female mice at 6 and 9–12 months compared with WT animals (**A**). The increase in p-S202/Thr205 tau correlated with Dkk-1 levels in 3xTg-AD mice (**C**) but not in WT mice (**B**). Representative Western blot and densitometric analysis data from 4–6 independent animals are shown. The data are presented as the mean ± SEM. (*p < 0.01, two-way ANOVA). Pearson correlation coefficient was applied for the Gaussian sample distribution

Because GSK3β is one of the main kinases implicated in tau phosphorylation, we measured the levels of the GSK3β-dependent phosphoepitope S202/Thr205 of tau protein. Although no significant changes in the total content of this epitope were observed in female WT mice during aging, an apparent reduction in the electrophoretic mobility of tau was observed, which could indicate an increase in total tau phosphorylation. Similarly, an apparent reduction of tau electrophoretic mobility was observed in 3xTg-AD mice, and this change was accompanied by a significant increase in the total p-S202/Thr205 tau concentration, particularly at 6 and 9–12 months, between the two strains (F_1,22_ = 19.91 *p* < 0.01) (Fig. 4A), To analyze the relationship between Dkk-1 levels and tau phosphorylation, we performed an index that revealed a significant (*R* = 0.54) correlation between Dkk-1 and tau phosphorylation in the transgenic mice but not in the WT mice (Fig. 4B, ). No correlation was observed between Dkk-1 levels and GSK3β activation in either the 3xTg-AD or WT during aging (data not shown), suggesting that additional factors may be involved in early GSK3β activation in female 3xTg-AD mice.

#### BDNF and pro-BDNF expression changes during aging and in the 3xTg-AD model

**Fig. 5:**
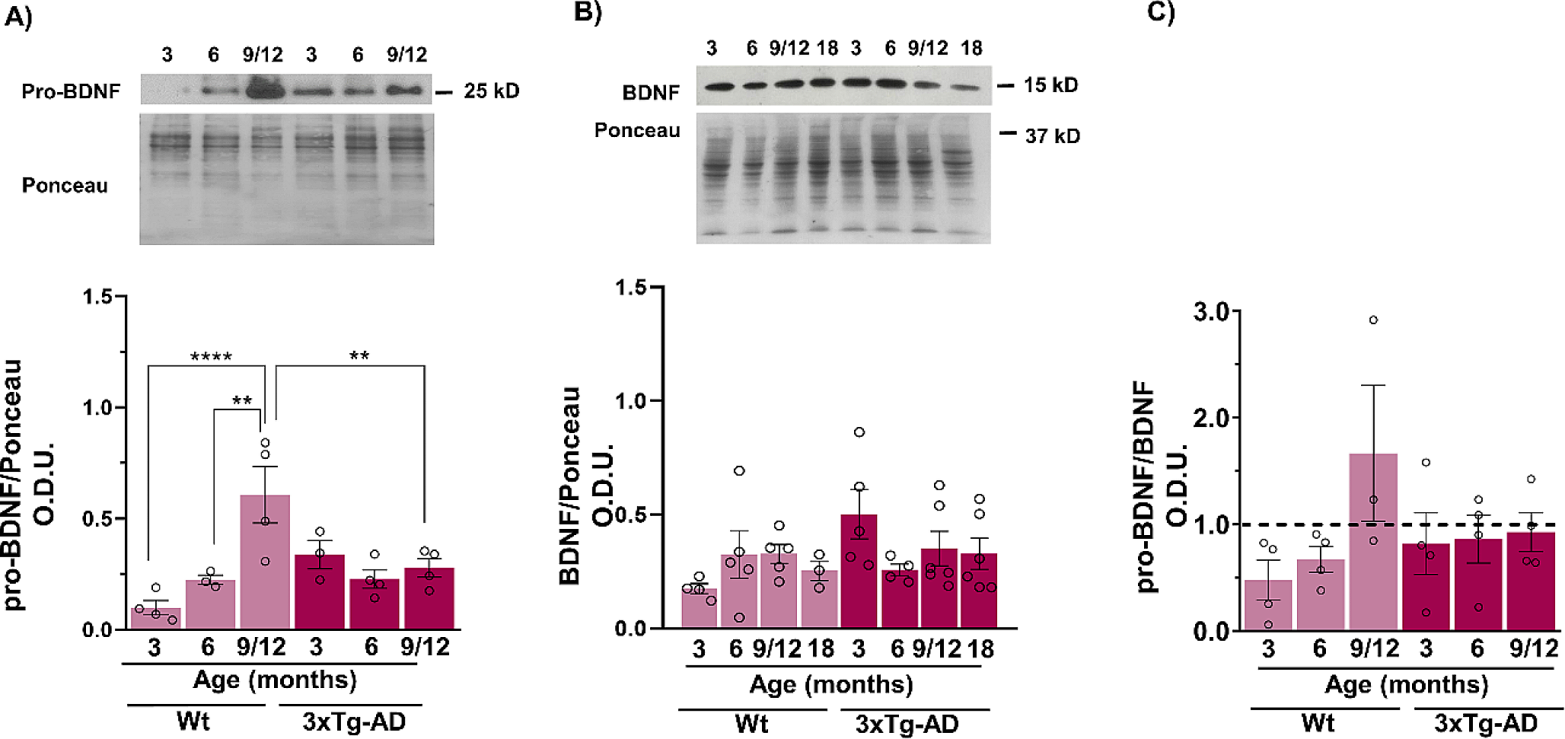
Changes in BDNF and pro-BDNF expression in the female hippocampus during aging and in the AD model. A progressive increase in pro-BDNF levels was observed at different ages in the WT mice (**A**), while in the 3xTg-AD mice, pro-BDNF levels were higher at 3 months and remained at almost the same level during aging. BDNF expression was higher in the 3xTg-AD mice than in the WT mice at 3 months (**B**). The pro-BDNF/BDNF ratio increased during aging in the WT animals and remained low in the 3xTg-AD mice (**C**). Representative Western blot and densitometric analysis data from 4–6 independent experiments are shown. The data are presented as the mean ± SEM. (**p < 0.01, ****p < 0.0001; two-way ANOVA)

BDNF has been implicated in neurodegenerative diseases, and its expression is regulated by the canonical Wnt pathway [9]. Thus, we analyzed whether the content of this neurotrophin changed in the hippocampi of WT and 3xTg-AD mice during aging. We observed a progressive increase in pro-BDNF levels throughout aging in the WT mice (F_2,16_ = 10.4, **p < 0.01, ****p < 0.0001), but not in the 3xTg-AD mice, in which the levels remained lower at all ages tested (Fig. 5A). Contrary to the pro-BDNF findings, no changes in the BDNF content were observed at any age or in any of the WT or transgenic mice (Fig. 5B). Interestingly, an increase in the proBDNF/BDNF ratio was observed during aging only in WT animals but not in 3XTg-AD animals (Fig. 5C).

### Dkk-1 and p-tau Levels are Significantly Greater in Females than in Males in the 3xTg-AD Model

**Fig. 6:**
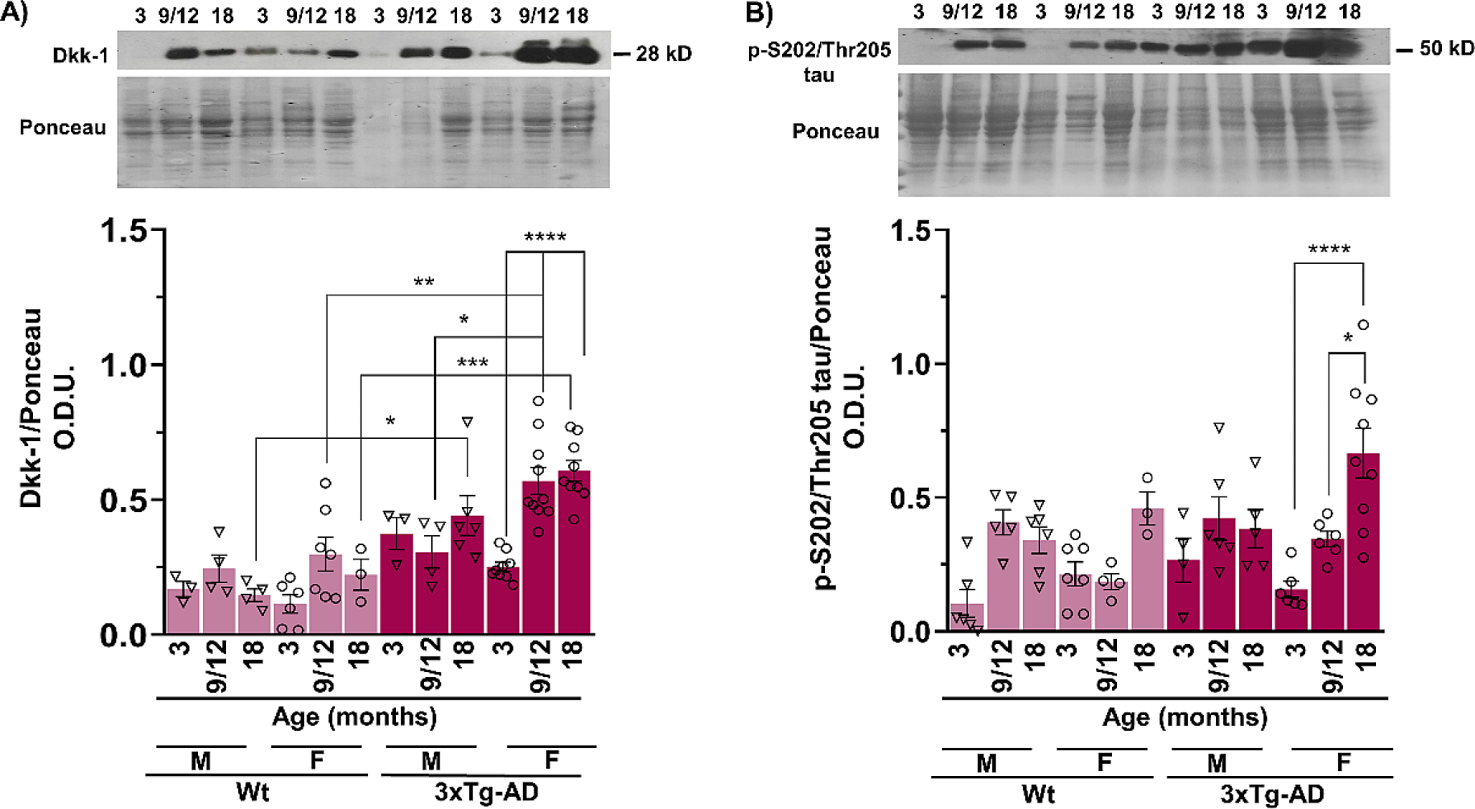
Sex-dependent differences in Dkk-1 and p-tau protein levels between WT and 3xTg-AD mice. Dkk-1 levels increased at all tested ages during the aging of both female (circles) and male (triangles) 3xTg-AD mice compared to WT mice of the same age and sex. Dkk-1 levels were higher in middle-aged female 3xTg-AD mice than in male 3xTg-AD mice (9–12 months old) (**A**). The pS202/Thr205-tau levels in both female and male WT and 3xTg-AD mice are shown in (**B**), and tau phosphorylation at this residue was more prominent in females than in males, particularly at advanced ages (9-12- and 18-month-old mice). Representative Western blot and densitometric analysis data from 3–10 independent experiments are shown. The data are presented as the mean ± SEM. (*p < 0.05, **p < 0.01, ***p < 0.001, ****p < 0.0001; three-way ANOVA)

Because sex-related differences in AD are more common and more severe in females than in males [32], we explored whether the increase in Dkk-1 levels differs between males and females in the WT and 3xTg-AD models with age. As expected, Dkk-1 was more highly expressed in female from both, WT and transgenic mice compared to males, particularly at middle age (9–12 months-old) (F_1,56_ = 3.822, *p* < 0.05), (Fig. 6A). In young transgenic mice (3 months-old), a trend was observed for lower levels of Dkk-1 in females than in males, suggesting a possible protective effect of female hormones, as has been previously reported in a model of cerebral ischemia and Dkk-1 expression [33]. While higher levels of Dkk-1 was observed in middle-aged and aged females compared to WT (F_1,56_ = 48.6, *p* < 0.01; F_1,56_ = 48.6, *p* < 0.001 ), in males, the differences among strains reached statistical significance up to 18 month-old (F_1,46_ = 48.6, *p* < 0.05). Consistent with Dkk-1 levels, a trend to increase tau hyperphosphorylation was observed at middle age in 3xTg-AD females compared to WT that continue progress at the advanced age of 18 month-old (Fig. 6B). The increase of tau hyperphosphorylation in transgenic female was highly significant since 9–12 and 18 months-old mice (F_2,55_ = 16.15, *p* < 0.05, *p* < 0.0001) Although a trend towards increased phosphorylated tau was observed in male animals regardless of the transgenic condition throughout aging, this increase did not reach statistically significant differences.

## Discussion

In this study, we found that Dkk-1 and Wnt7a are differentially expressed during normal aging (in WT mice) compared to pathological aging (in 3xTg-AD mice). Aging was accompanied by a gradual increase in Dkk-1 in WT mice, which was strongly exacerbated since young in 3xTg-AD mice correlating with early activation of the kinase GSK3β before tau phosphorylation. Dysregulation of Wnt signaling molecules have been reported in postmortem tissue from AD patients, suggesting that the pathway is impaired at late stages of the disease [7, 28]. Similarly, it has been reported that inhibition of the canonical Wnt pathway triggers the appearance of AD hallmarks in vitro [11, 12, 28] and in vivo [34, 35]. However, the questions of whether in vivo Wnt dysregulation occurs prior to the appearance of disease hallmarks and whether this dysregulation is a consequence or cause of Wnt signaling changes remain open. Herein, we found that the canonical Wnt pathway seems to be impaired beginning in the early stages of pathology prior to the accumulation of p-S202/Thr205-tau, one of the hallmarks that defines AD pathology. Later, during the progression of the pathology, we also found a decrease in the levels of the canonical agonist Wnt7a and at more advances ages, the coreceptor LRP6. Although these changes also occur during normal aging, as reported in other aged species [26, 27], in the 3xTg-AD mice, the downregulation of these Wnt components occurred earlier than in the control mice and was particularly severe. In addition to the negative effects of Dkk-1 and the decrease in the agonist Wnt7a, we found that the symptomatic stage of pathology in transgenic mice could worsen synaptic function because of its role in promoting synaptic plasticity events [36, 37].

The mechanisms involved in Dkk-1 upregulation during aging and pathology are not known; however, Dkk-1 transcription is modulated by the tumor suppressor protein p53, which responds to cellular damage [38]. Thus, the signals that may promote the early accumulation of Dkk-1 in transgenic mice prior to the pathological accumulation of p-tau can be associated with the induction of several biochemical stressors that occur early in pathology but that only appear in WT mice at more advanced ages. Another mechanism underlying the early upregulation of Dkk-1 may be associated in part with the activation of the c-Jun N-terminal kinase (JNK) pathway, which senses harmful stimuli in cells, such as oxidative stress [39]. Accordingly, JNK has been found to be activated in human postmortem AD brain samples [40] and in presymptomatic stages in 2.5-month-old 3xTg-AD mice [41]. In the brains of young 3xTg-AD mice, the activation of JNK could be triggered by several stressors, such as reactive oxygen species, as mentioned above, by hypoxia, and by the early presence of oligomeric forms of Aβ [42]. Notably, the upregulation of Dkk-1 revealed important sexual dimorphism in the WT and AD model. The differences were marked according to animal age: there was a trend toward lower Dkk-1 content in young females but a greater increase in Dkk-1 in old females than in males, suggesting a lack of protective female hormone effects. Sexual dimorphism is gaining interest, as clinical data show that women are at greater risk of developing AD and having worse pathology and faster cognitive decline. In this sense, the impact of female hormones, such as progesterone, on Dkk-1 expression was reported [43]. It was also found that 17β-estradiol downregulates Dkk-1 in a model of global cerebral ischemia [33]. Interestingly, compared with WT females, 3xTg-AD females are known to have a shorter reproductive window, which could suggest that transgenic females experience an early decrease in the neuroprotective effects of estrogens and progesterone on Dkk-1 expression. Unlike women, female rodents do not experience menopause; however, between the ages of 11 and 16 months, they undergo a transition period characterized by an altered estrous cycle [44], corresponding to the age at which Dkk-1 upregulation was prominent in the female WT mice.

Notably, female 3xTg-AD mice displayed more prominent amyloid plaques, neurofibrillary tangles, neuroinflammation, and spatial cognitive deficits than male 3xTg-AD mice, as has been widely documented [45–47]. These differences may explain the lower content of p-tau observed in male 3xTg-AD model mice.

Although the 3xTg-AD model has the advantage that pathology occurs in an age-dependent manner, some of the molecules in the Wnt pathway that are altered and the expression of the hallmark p-tau that are commonly observed in females can be associated with the presence of the Thy1 promoter transgene cassette for mutated APP and tau. This promoter contains an estrogen response-like element that, as suggested, can explain the higher expression of transgenes in females [47, 48]. However, more research is needed to fully explain these sex differences because the age-associated increases in the Dkk-1 and p-tau levels in aged females were maintained at the age at which estrogen levels tend to decline.

As mentioned above, we identified 2 impairments in the Wnt signaling pathway throughout the pathology. The second impairment was the downregulation of the Wnt7a agonist and the coreceptor LRP6 that occurred in the symptomatic stage of the pathology in the 3xTg-AD mice but also occurred in the older WT animals. Recent research has shown that Wnt signaling activation can also be regulated by signalosome endocytosis of the Wnt receptor complex [49]. Notably, Dkk-1 induces the internalization of LRP6 together with the protein Kremen, inhibiting the canonical Wnt pathway [50] and promoting LRP6 degradation. This mechanism supports a model in which the upregulation of Dkk-1 during aging and incipient AD-like pathology negatively regulates Wnt signaling and subsequently reduces the content of the LRP6 coreceptor, as we found here, worsening the Wnt molecular pathway that participates in synapse assembly and function in the mature brain [51, 52]. GSK3β is considered an important player in aging studies because it is a central node for the control of multiple pathways involved in cellular metabolism, growth, survival, inflammation, and senescence, among others [24, 53, 54]. As part of the first impairment in the Wnt signaling pathway reported in this study, enhanced GSK3β kinase activation was found beginning at the presymptomatic stage of the pathology in 2-3-month-old 3xTg-AD mice, similar in magnitude to what occurred until 9–12 months of age in the WT animals, correlating with the induction of the Wnt antagonist Dkk-1. These observations point to a role of Dkk-1 in inducing the hallmark of AD, at least through the phosphorylation of tau in the 3xTg-AD model. In agreement with the present results, the activity of GSK3β was found to increase prior to the formation of nurofibrillary tangles in the brains of AD patients [55], and the overexpression of this kinase was also found in the prefrontal cortex and was associated with cognitive decline in AD patients [7]. Taken together, these results reveal important changes in GSK3β activity in middle-aged WT animals, which were significantly exacerbated in young 3xTg-AD mice, suggesting a cascade of events that starts with Dkk-1 overproduction as a key factor associated with the development of AD pathology.

Although the BDNF gene promoter contains binding motifs for Wnt-dependent TCF/LEF transcription factors [9], we did not observe significant changes in the protein content of this neurotrophin in the WT or in the 3xTg-AD mice, in agreement with other works in which 3xTg-AD mice were found to have BDNF levels that are comparable to those of WT controls [56]. However, a significant increase in pro-BDNF levels was observed in WT animals during aging, while BDNF levels remained stable relative to those in young adults according to previous work [57, 58]. These results suggest that changes in gene expression do not correlate with changes in protein levels or that abnormalities in the production of mature BDNF or other components of the BDNF signaling pathway, such as TrkB or p75NTR, may be involved in aging and AD pathology.

## Conclusion

Previous evidence has suggested a role of Wnt dysregulation in brain aging and in AD. Our findings indicate that the age-dependent increase in Dkk-1 levels was exacerbated in both male and female transgenic mice; in these female mice, the Dkk-1 levels were correlated with the p-tau levels. These results support the idea that reactivation of the Wnt pathway may be considered a therapeutic strategy for improving brain function during aging and for delaying, to some extent, the occurrence of some neuropathological changes in AD.

## Data Availability

No datasets were generated or analysed during the current study.
